# Angioimmunoblastic T‐cell lymphoma contains multiple clonal T‐cell populations derived from a common *TET2* mutant progenitor cell

**DOI:** 10.1002/path.5376

**Published:** 2020-01-16

**Authors:** Wen‐Qing Yao, Fangtian Wu, Wenyan Zhang, Shih‐Sung Chuang, Joe S Thompson, Zi Chen, Shao‐Wei Zhang, Alexandra Clipson, Ming Wang, Hongxiang Liu, Hani Bibawi, Yuanxue Huang, Luis Campos, John W Grant, Penny Wright, Hesham EI‐Daly, Lívia Rásó‐Barnett, Lorant Farkas, George A Follows, Zifen Gao, Ayoma D Attygalle, Margaret Ashton‐Key, Weiping Liu, Ming‐Qing Du

**Affiliations:** ^1^ Division of Cellular and Molecular Pathology, Department of Pathology University of Cambridge Cambridge UK; ^2^ Department of Pathology West China Hospital, Sichuan University Chengdu PR China; ^3^ Department of Haematology Jiangsu Province Hospital, Nanjing Medical University Nanjing PR China; ^4^ Department of Pathology Chi‐Mei Medical Center Tainan Taiwan; ^5^ Department of Haematology Addenbrooke's Hospital, Cambridge University Hospitals NHS Foundation Trust Cambridge UK; ^6^ Molecular Malignancy Laboratory Addenbrooke's Hospital, Cambridge University Hospitals NHS Foundation Trust Cambridge UK; ^7^ Department of Histopathology Addenbrooke's Hospital, Cambridge University Hospitals NHS Foundation Trust Cambridge UK; ^8^ The Haematopathology and Oncology Diagnostic Service Addenbrooke's Hospital, Cambridge University Hospitals NHS Foundation Trust Cambridge UK; ^9^ Department of Pathology Health Science Centre, Peking University Beijing PR China; ^10^ Histopathology Department The Royal Marsden Hospital London UK; ^11^ Department of Cellular Pathology Southampton University Hospitals National Health Service Foundation Trust Southampton UK

**Keywords:** AITL, clonality, *TET2* mutation, progenitor cells, lymphoma genesis

## Abstract

Angioimmunoblastic T‐cell lymphoma (AITL) is a neoplastic proliferation of T follicular helper cells with clinical and histological presentations suggesting a role of antigenic drive in its development. Genetically, it is characterized by a stepwise acquisition of somatic mutations, with early mutations involving epigenetic regulators (*TET2*, *DNMT3A*) and occurring in haematopoietic stem cells, with subsequent changes involving signaling molecules (*RHOA*, *VAV1*, *PLCG1, CD28*) critical for T‐cell biology. To search for evidence of potential oncogenic cooperation between genetic changes and intrinsic T cell receptor (TCR) signaling, we investigated somatic mutations and T‐cell receptor β (TRB) rearrangement in 119 AITL, 11 peripheral T‐cell lymphomas with T follicular helper phenotype (PTCL‐TFH), and 25 PTCL‐NOS using Fluidigm polymerase chain reaction (PCR) and Illumina MiSeq sequencing. We confirmed frequent *TET2*, *DNMT3A*, and *RHOA* mutations in AITL (72%, 34%, 61%) and PTCL‐TFH (73%, 36%, 45%) and showed multiple *TET2* mutations (2 or 3) in 57% of the involved AITL and PTCL‐TFH. Clonal TRB rearrangement was seen in 76 cases with multiple functional rearrangements (2–4) in 18 cases (24%). In selected cases, we confirmed bi‐clonal T‐cell populations and further demonstrated that these independent T‐cell populations harboured identical *TET2* mutations by using BaseScope *in situ* hybridization, suggesting their derivation from a common *TET2* mutant progenitor cell population. Furthermore, both T‐cell populations expressed CD4. Finally, in comparison with tonsillar TFH cells, both AITL and PTCL‐TFH showed a significant overrepresentation of several TRB variable family members, particularly TRBV19*01. Our findings suggest the presence of parallel neoplastic evolutions from a common *TET2* mutant haematopoietic progenitor pool in AITL and PTCL‐TFH, albeit to be confirmed in a large series of cases. The biased TRBV usage in these lymphomas suggests that antigenic stimulation may play an important role in predilection of T cells to clonal expansion and malignant transformation. © 2019 The Authors. *The Journal of Pathology* published by John Wiley & Sons Ltd on behalf of Pathological Society of Great Britain and Ireland.

## Introduction

Patients with angioimmunoblastic T‐cell lymphoma (AITL) often present with clinical and laboratory autoimmune features 1. Histologically, the lymphoma is characterised by a polymorphous infiltrate with the neoplastic T cells typically forming clusters in the vicinity of prominent arborising high endothelial venules and expanded follicular dendritic cells 2. The lymphoma cells originate from T follicular helper (TFH) cells and possess their cardinal phenotype, albeit showing variable CD10, CXCL13, ICOS, PD‐1, and BCL6 expression. The lymphoma cells also preserve the major function of TFH cells, for example, helping B cells in their antibody production 3, 4. Because high‐affinity TCR is essential for the commitment of CD4+ T cells to differentiate into TFH cells as well as their maintenance and survival, active TCR signaling most likely plays an important role in the pathogenesis of AITL. This is also supported indirectly by the finding of a number of somatic genetic changes, which involve molecules downstream of TCR signaling.

Although exome and targeted sequencing have identified a wide spectrum of genetic changes in AITL, and also demonstrated a remarkable similarity in the mutation profile between AITL and peripheral T‐cell lymphoma (PTCL) with a TFH cell phenotype, suggesting their close relationship 5, 6, 7, 8, 9, 10, 11, 12. In addition, these studies revealed distinct classes of genetic changes that occur at different stages of AITL development.

Class I genetic changes include mutations in epigenetic (DNA methylation) regulators namely *TET2*, *DNMT3A*, and *IDH2*, with the *TET2* gene frequently affected by more than one mutation 7, 13, 14, 15, 16, 17. Mutations in these genes are found in a range of haematological malignancies with *IDH2* mutation additionally seen in several types of solid tumours 15, 18, 19, 20. In patients with AITL, the lymphoma associated *TET2* and *DNMT3A* mutations most likely occur at an early stage of haematopoiesis, as they are also observed in several lineages of non‐neoplastic cells including non‐neoplastic B and CD8+ T cells 7, 13, 14, 16, 21, 22. Thus, *TET2* and *DNMT3A* mutations are initiating events, promoting ‘clonal haematopoiesis’ and increasing the risk of lymphomagenesis 10, 23, 24.

Class II genetic changes include mutation in *RHOA*, *VAV1*, *PLCG1*, and *CD28*, as well as the *CTLA4‐CD28, ITK‐SYK*, and *VAV1‐STAP2* fusion 6, 7, 10, 17, 25, 26, 27, 28. These genetic changes are secondary events, and they involve molecules critical for the biology of T cells, thus most likely promoting malignant transformation and clonal expansion, consequently generating the malignant phenotype of AITL.

Among the above genetic changes, mutations in *TET2*, *DNMT3A*, and *RHOA* are highly frequent and often concurrent in AITL, arguing for their potential cooperation in lymphoma development. This is supported by several mouse model studies, which demonstrate oncogenic cooperation between *TET2* inactivation and *DNMT3A* mutation 29, and also between *TET2* inactivation and *RHOA* mutation 30, 31. It is pertinent to speculate that these genetic changes may also cooperate with the intrinsic TCR signaling in clonal evolution and malignant transformation. To search for such evidence, we investigated TCR gene usage and somatic mutations in 155 cases of AITL and PTCL by targeted sequencing. Our findings suggest the presence of multiple independent neoplastic evolutions from a common *TET2* mutant haematopoietic progenitor pool in AITL and PTCL‐TFH.

## Materials and methods

### Tissue materials and DNA extraction

The use of archival tissues for research was approved by the ethics committees of the involved institutions.

A total of 155 cases of AITL and PTCL were successfully investigated and they were from Department of Pathology, West China Hospital, Chengdu, PR China (n = 91); Department of Histopathology, Addenbrooke's Hospital, Cambridge, UK (n = 32); Department of Cellular Pathology, Southampton University Hospitals, Southampton, UK (n = 21); Department of Pathology, Chi‐Mei Foundation Hospital, Taiwan (n = 8); and Department of Histopathology, Royal‐Marsden Hospital, UK (n = 3). Formalin‐fixed paraffin‐embedded diagnostic tissue biopsies were available in each case, and additionally fresh frozen specimens and high molecular weight (HMW) DNA samples were available in nine cases of AITL that were previously investigated by whole exome sequencing 10, with 92% of these specimens being lymph node biopsies. The histology and immunophenotype of these cases were reviewed by specialized haematopathologists, where necessary additional immunohistochemistry for CD4 (mouse monoclonal 4B12, Leica Biosystems, Milton Keynes, UK), CD10 (mouse monoclonal 56C6, Leica Biosystems), PD1 (mouse monoclonal NAT105, Sigma, Gillingham, UK), BCL6 (mouse monoclonal LN22, Leica Biosystems), and CXCL13 (mouse polyclonal, R&D Systems, Abingdon, UK) was performed using an automatic Bond‐III platform (Leica Biosystems). The final lymphoma diagnoses were established according to the 2016 WHO classification of tumours of haematopoietic and lymphoid tissues 2, and they included 119 AITL, 11 PTCL‐TFH (including one follicular T‐cell lymphoma), and 25 PTCL‐NOS.

DNA was extracted from enriched tumour cell population (>30%) and where possible non‐neoplastic cells by crude microdissection using the QIAamp DNA‐MicroKit. The quality of DNA from tumour cells was assessed by PCR and those showing amplification of ≥300 bp genomic fragments were used for mutation screening by targeted sequencing 32, while those from non‐neoplastic cells were used to exclude germline variants.

### Targeted sequencing using Fluidigm Access Array and Illumina MiSeq

This was performed as described previously with each of the DNA samples analysed in duplicate for mutations in *TET2*, *DNMT3A*, *IDH2*, *RHOA*, *PLCG1*, *CCND3*, *CD28*, and *TNFRSF21* 32. In brief, genomic DNA (50 ng) was pre‐amplified with a cocktail of all primer pairs to enrich targets, then subjected to Fluidigm Access Array multiplex PCR (up to four primer pairs combined), and followed by barcoding and Illumina MiSeq sequencing 32. Primers and PCR conditions are described in supplementary material, Table S1.

For single nucleotide variant (SNV) detection, bam files were processed using a pipeline based on GATK v3.6 best practices including indel realigned and recalibration steps. The calling variant was run using UnifiedGenotyper with 10 000 to prevent downsampling 33, 34. As UnifiedGenotyper was unable to call SNVs at <8% AAF (alternative allele frequency) reliably, MuTect2 was additionally employed for detection of hotspot mutations at low AAF values (down to 2%). Indel detection was separately carried out on the recalibrated bam files using Pindel v0.2.5 35. Variant call files were concatenated to produce one library vcf each for the SNV and Indel pipelines. These library files were then filtered using a combination of vcftools v0.1.15 and bedtools v2.25 for read depth, quality score, and known PCR/sequence artefacts. Further filtering was accomplished using an in‐house script to remove variants in intronic regions outside essential splicing sites, SNPs with a minor allele frequency ≥ 1% (1000 Genomes Project, European super population) and synonymous changes. In addition, missense variants predicted to be benign by seven or more of nine functional prediction tools (SIFT, Polyphen2 HDIV, Polyphen2 HVAR, LRT, MutationTaster, MutationAssessor, FATHMM, SVM score, and LR score) were excluded. The resulting novel variants were further scrutinized by reviewing the bam file to eliminate any potential PCR/sequence artefacts. Only the variants that appeared in both replicates, with novel changes ≥8% AAF and known hotspot changes ≥2% AAF, were regarded as a true change. Where possible, variants were confirmed as somatic by using Sanger sequencing analysis of corresponding non‐neoplastic DNA or assumed to be somatic if previously confirmed as somatic in the COSMIC database.

### Sequencing analysis of the rearranged TCR genes using Illumina MiSeq

The rearranged TRB (T‐cell receptor beta) genes were amplified using the BIOMED‐2 protocols and then sequenced with Illumina MiSeq platform. In brief, genomic DNA (10 and 20 ng) was amplified in duplicate for the rearranged TRB genes (TCRB tube A and B) according to the BIOMED‐2 protocols with the exception that the primers were tagged with a common sequence (CS1: 5'‐ACACTGACGACATGGTTCTACA‐3′, or CS2:5'‐TACGGTAGCAGAGACTTGGTCT‐3′) 36. The amplified products were routinely purified using AMPure XP beads (Beckman Coulter, UK), barcoded and pooled for Illumina MiSeq sequencing as described previously 32. The sequences obtained were analysed using the online software VIDJIL 37, and only productive rearrangements were included in further analyses to search for evidence of biased TCR gene usage in the T‐cell lymphoma entities investigated. The clonal size was calculated for each unique VDJ rearrangement as percentage of the total TRB sequence reads from the corresponding PCR tube, and the mean between the two replicates was used for data presentation, with ≥10% reads used as cutoff value for defining clonal TRB rearrangement.

### BaseScope *in situ* hybridization (ISH)

On selected cases, specific DNA probes were designed to target the unique TRB VDJ junctional sequence or *TET2* mutations and used for ISH to visualize the corresponding clonal T cells and the cells carrying the *TET2* mutation, respectively. The BaseScope ISH was carried out according to the manufacturer's instructions (Advanced Cell Diagnostics, Newark, CA, USA). In brief, formalin‐fixed paraffin embedded tissue sections were routinely dewaxed, treated with targeted retrieval reagents at 100 °C for 15 min in a steamer, and digested with protease IV for 15 min at 40 °C in a hybridization oven. The slides were hybridized with a BaseScope probe for 2 h at 40 °C, followed by a serial amplification steps at 40 °C in a hybridization oven and at room temperature (the last two amplification steps), and finally incubated with the Fast Red substrate at room temperature to visualise the hybridization signals.

For double BaseScope ISH, the TRB‐VDJ and *TET2* mutation probes were labeled in different ‘channels,’ allowing independent signal amplification, with the TRB‐VDJ signal detected using Fast Red and the *TET2* mutation signal using the kit's Green reagents. The double ISH was similarly performed using the BaseScope Duplex detection kit according to the manufacturer's instructions.

For TRB‐VDJ and CD4/CD8 double staining, TRB‐VDJ BaseScope ISH was performed as described earlier, and the slide was then routinely stained with a CD4 or CD8 antibody using a Bond‐III platform with Bond Polymer Refine Detection reagents (Leica Biosystems).

### Statistical analysis

Fisher's exact test was used to examine potential associations between categorical variables. A chi‐square with Yate's correction test was used to compare the TRBV usage between AITL/PTCL‐TFH and normal tonsillar follicular helper T cells 38, and between PTCL‐NOS and peripheral blood T cells (a total of 22 704 rearranged TRB sequences from 550 healthy donors) 39.

## Results

### Mutation profile in AITL, PTCL‐TFH, and PTCL‐NOS

**Figure 1:**
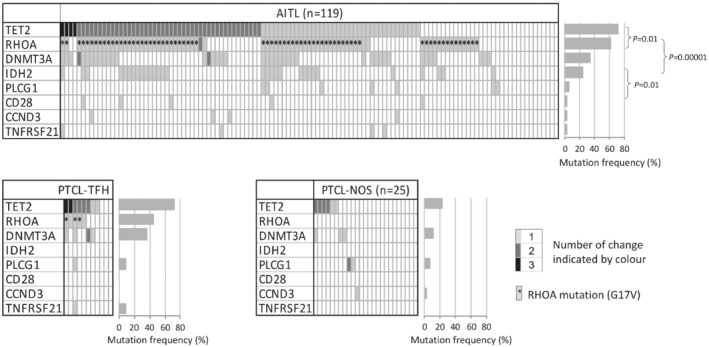
Mutation profile in AITL, PTCL‐TFH, and PTCL‐NOS.

In general, the mutation profile was broadly similar between AITL and PTCL‐TFH, with the most frequent mutation being *TET2* (72%, 73% respectively), followed by *RHOA* (61%, 45% respectively) and *DNMT3A* (34%, 36% respectively) (Figure 1). *IDH2* and *CD28* mutations were seen in only AITL (25%, 4% respectively), and not in PTCL‐TFH, with mutation in the remaining genes being at low frequencies in both groups. In contrast, PTCL‐NOS had a much lower frequency of mutation in *TET2* (24%) and *DNMT3A* (12%), and no mutation in *RHOA*, *IDH2*, and *CD28*.

Correlation analysis demonstrated a significant association between *TET2* and *RHOA* (*P* = 0.01), *RHOA* and *IDH2* (*P* = 0.00001), and *IDH2* and *CD28* (*P* = 0.01) mutation in AITL. Overall, there was a tendency of mutual exclusion among *PLCG1*, *CD28*, *CCND3*, and *TNFRSF21* mutations.

### Mutation characteristics

Because the mutation profile was broadly similar between AITL and PTCL‐FTH, the characteristics of mutations from both groups were presented together below (supplementary material, Figures S1 and S2, Table S2).

#### *TET2* mutation

A total of 154 *TET2* mutations were seen in 94 cases of AITL and PTCL‐FTH, and 118 (77%) of these mutations were frameshift indels or nonsense changes, and rather widely distributed, predicting variably truncated protein products (supplementary material, Figure S1). A small proportion of the mutations were missense changes and they were clustered in the cysteine rich and DSBH (double‐stranded β helix) domains, which were essential for the integrity of the overall structure and the catalytic activity of TET2 40. These mutational features are similar to those previously reported 6, 7.

Multiple *TET2* mutations ranging from 2 and 3 were seen in 54 cases, accounting for 57% of the cases investigated. Among the cases with multiple *TET2* mutations, 31 cases had two deleterious changes (frameshift indels, nonsense changes, or mutation at the essential splicing site), further highlighting potential inactivation of both *TET2* alleles.

#### *DNMT3A* mutation

Sixty percent of the *DNMT3A* mutations identified were missense changes that occurred nearly exclusively in the methyltransferase domain, with R882 being a hotspot accounting for 25% of all mutations (supplementary material, Figure S1). The remaining mutations were frameshift indels, nonsense changes, and substitutions at the essential splicing site, which were widely distributed (supplementary material, Figure S1).

#### *RHOA* mutation

This was overwhelmingly dominated by changes in G17, with G17 V being the most frequent, accounting for 91% of all *RHOA* mutations (supplementary material, Figure S1). The remaining mutations were S26R and C20W, each seen in one case. The majority of cases with *RHOA* mutation had *TET2* mutation (Figure 1), underpinning their oncogenic cooperation in AITL development 30, 31.

*IDH2* mutation occurred exclusively in R172, with R172G being the most frequent change, and the majority of cases with *IDH2* mutations also had both *TET2* and *RHOA* mutations (Figure 1 and supplementary material, Figure S1). IDH R172 mutant confers a neomorphic enzymatic activity, capable of converting α‐ketoglutarate (αKG) to the D form of 2 hydroxyglutarate (2HG), which inhibits TET and Jumonji (JMJ) family histone demethylases, thereby promoting lymphomagenesis 21, 41.

*PLCG1* mutation was seen in eight cases, with the missense changes including R48W, S345F, G869E, and D1165H being reported previously and shown to be gain of function changes (supplementary material, Figure S1) 17, 25.

*CD28* mutation was observed in five cases, with four being missense changes at T195 in its cytoplasmic domain (supplementary material, Figure S1). These mutations have been shown to be an activating change, enhancing CD28 downstream signaling 17, 27.

*CCND3* mutation was found in four cases, with three mutations including two nonsense, one frameshift indel, and P284S at the C‐terminal region downstream of the Cyclin‐C domain, which was a mutation hotspot (supplementary material, Figure S1) 42, 43. Mutations in this C‐terminal region affect a phosphorylation motif important for polyubiquitination and proteasome‐mediated degradation, consequently enhancing CCND3 stability and its function 44.

### High mutation allele frequency in *TET2* and *DNMT3A*

In support of the previous observations that *TET2* and *DNMT3A* mutations occur at an early stage of haematopoiesis, and were seen in non‐neoplastic B and CD8+ T cells in patients with AITL 7, 13, 14, 15, 16, we found that the mutation allele frequencies of both *TET2* and *DNMT3A* were significantly higher than those of *RHOA* and *IDH2* (supplementary material, Figure S3). In contrast, there was no difference in the mutation allele frequencies between *TET2* and *DNMT3A*, nor between *RHOA* and *IDH2* (supplementary material, Figure S3). Of interest, the mean age of patients with *TET2, RHOA*, *DNMT3A*, or *TNFRSF21* mutation was significantly higher than those without *TET2* (66 versus 60 years, *P* = 0.003), *RHOA* (66 versus 61, *P* = 0.01), *DNMT3A* (68 versus 62 years, *P* = 0.001), or *TNFRSF21* (77 versus 63, *P* = 0.01) mutation respectively.

### Evidence of more than one clonal T‐cell population

**Figure 2:**
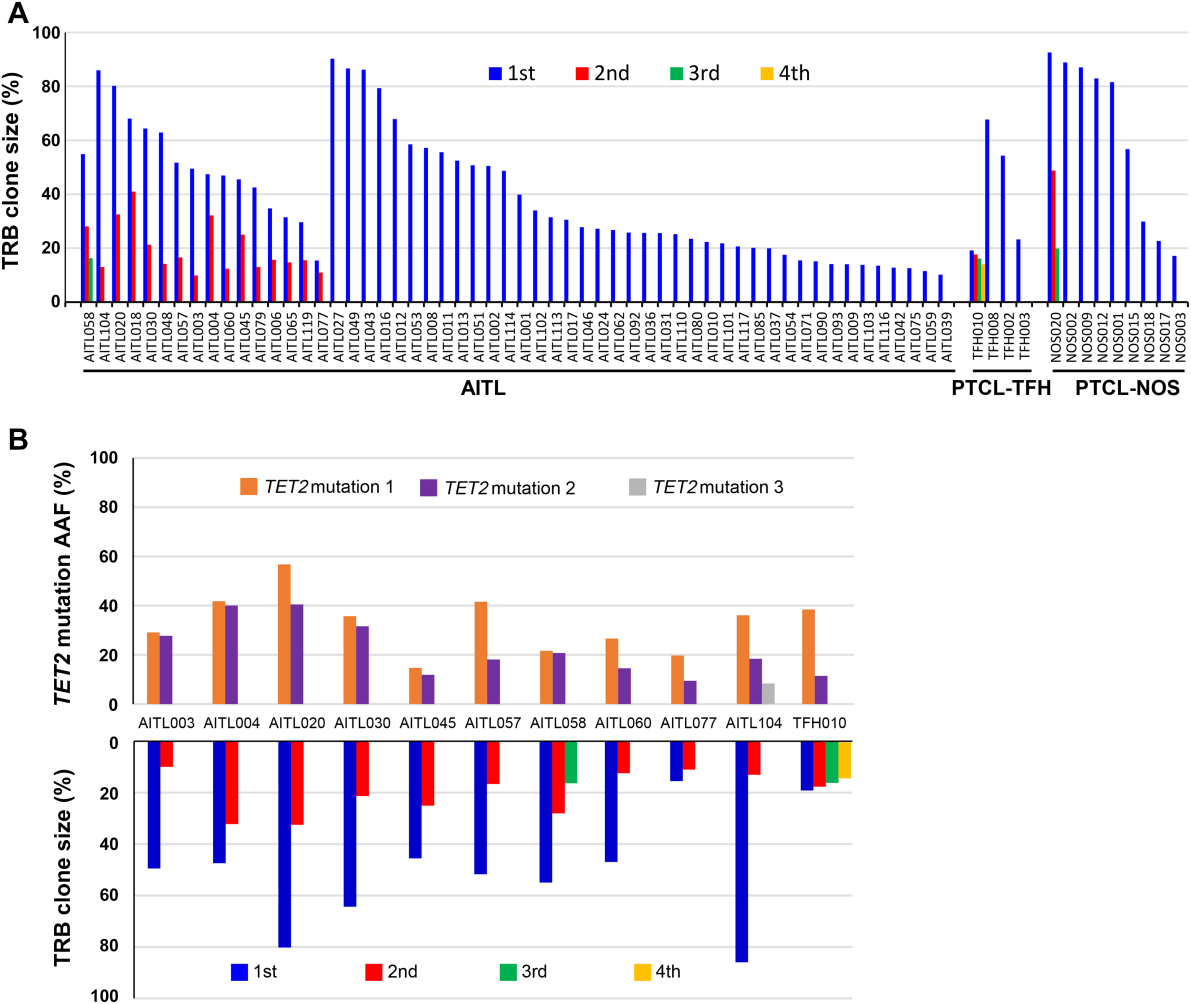
TRB functional rearrangements in AITL, PTCL‐TFH, and PTCL‐NOS. (A) Number of functional TRB rearrangements and their clonal sizes. (B) Comparison between the loads of *TET2* mutations and the size of clonal TRB rearrangements on select cases.

A total of 112 cases were successfully investigated for TRB rearrangements by the BIOMED‐2 TRB PCR (tube A and B) and Illumina MiSeq sequencing, 76 (68%) of these cases yielded a clonal result using ≥10% as a cut‐off. Of the 76 informative cases, 69 cases (62%) showed functional clonal TRB rearrangements, and they included 56 AITL, 4 PTCL‐TFH and 9 PTCL‐NOS. Multiple functional TRB rearrangements (2–4) were seen in 18 cases (26%) including 16 AITL, one PTCL‐TFH, and one PTCL‐NOS (Figure 2 and supplementary material, Figure S4). The number of cases with multiple functional TRB rearrangements was much higher in cases with multiple *TET2* mutations (11/13 = 85%) than those with a single *TET2* mutation (21/38 = 55%), albeit the difference was not statistically significant. Among these cases, one case (PTCL‐TFH010), which harboured two *TET2* and two *DNMT3A* mutations, had four functional TRB rearrangements. These observations suggest possible bi‐ or oligo‐clonal T‐cell populations rather than bi‐allelic functional rearrangements (Figure 2).

### Confirmation of two clonal T‐cell populations that carry the same *TET2* mutations

**Figure 3:**
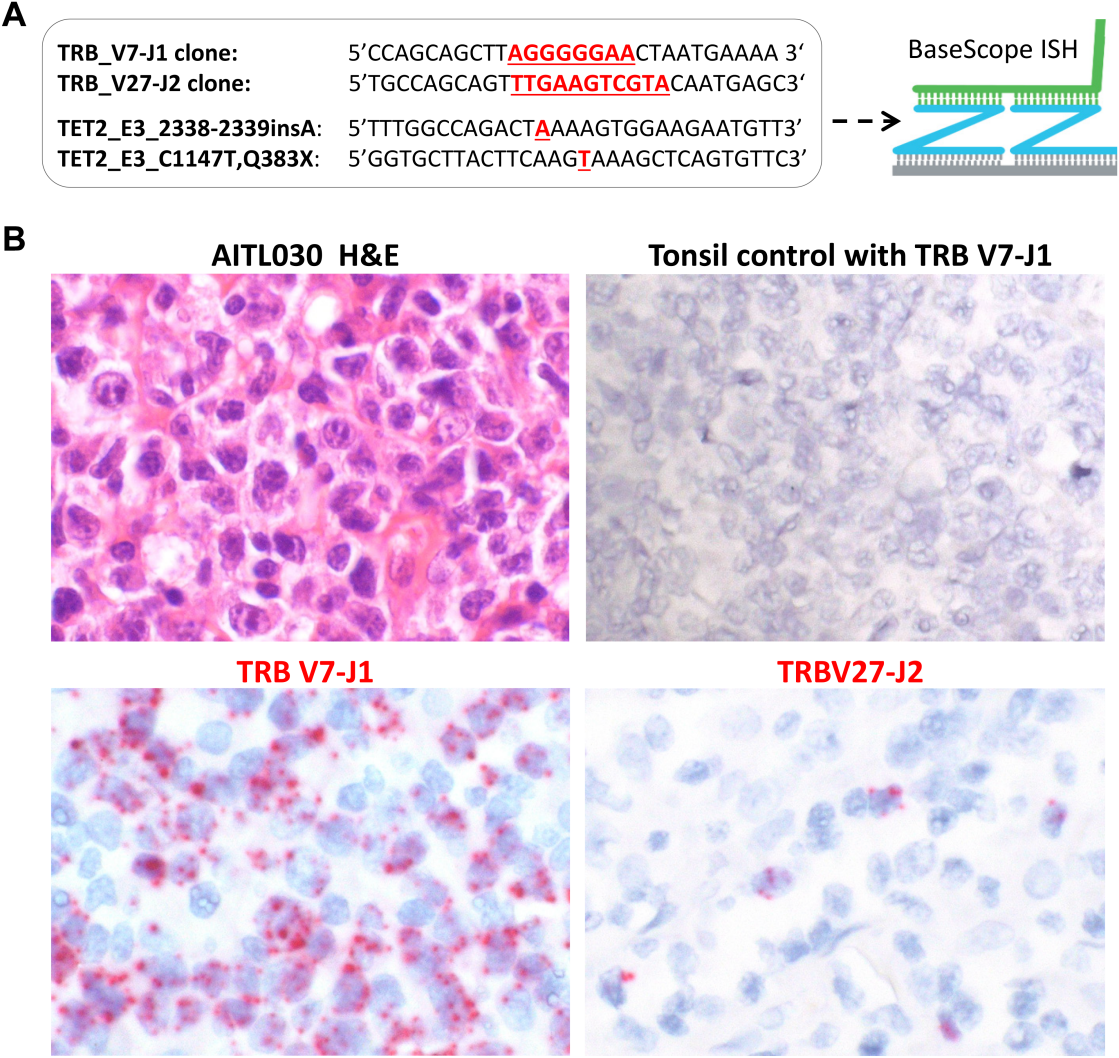
Confirmation of two clonal T‐cell populations by BaseScope ISH with TRB clone specific probes. (A) BaseScope probe design with the unique VDJ junctional sequence and *TET2* mutation highlighted in red. (B) BaseScope *in situ* hybridization in case AITL030 with tonsil serving as a negative control. The TRB V7‐J1 probe identifies a diffuse cell population, whereas the TRB V27‐J2 probe reveals only scattered cells, confirming that they represent two independent clonal T‐cell populations.

**Figure 4:**
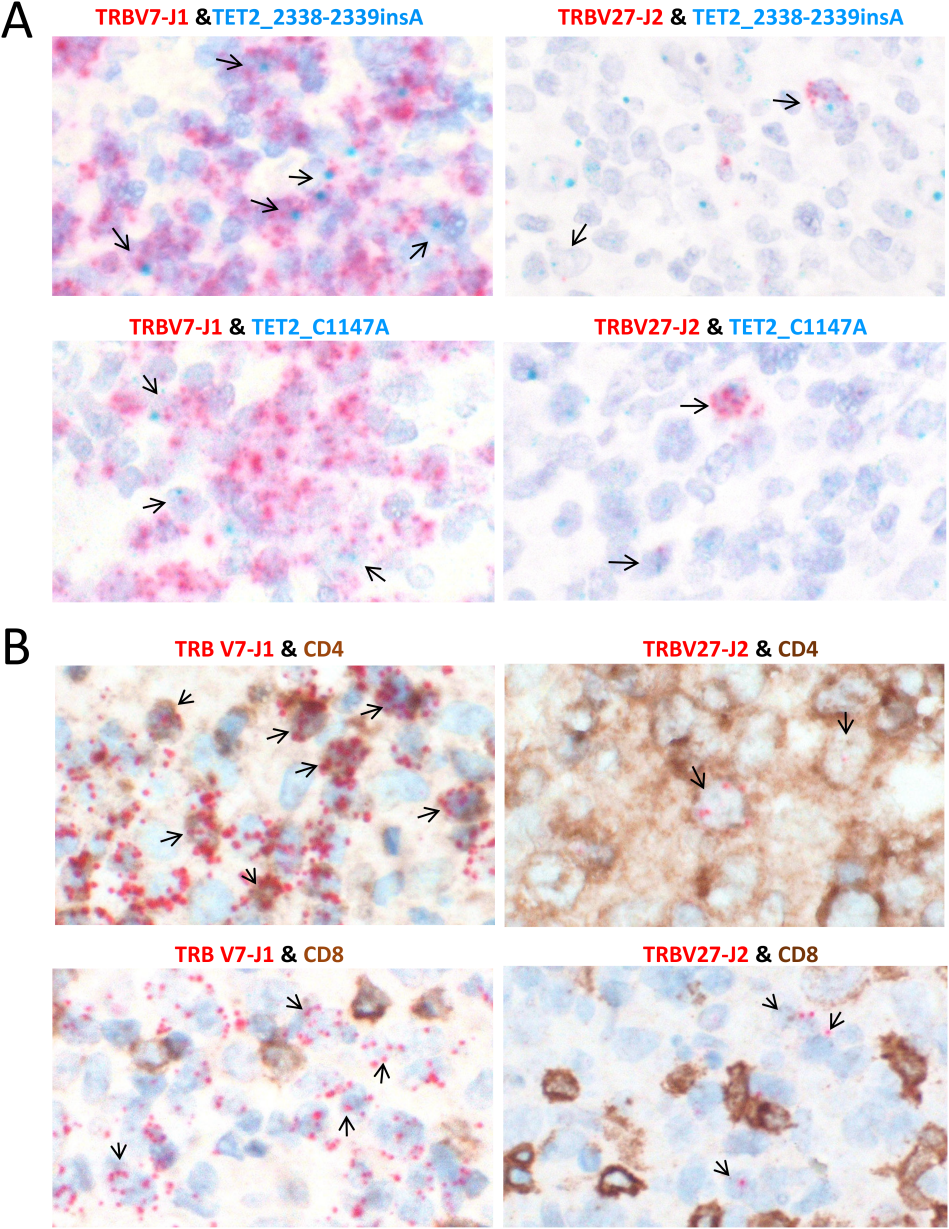
Demonstration of *TET2* mutations and CD4 positivity in two clonal T‐cell populations of AITL030. (A) Double BaseScope *in situ* hybridization shows the presence of both *TET2* mutations in the two clonal T‐cell populations of AITL030. Please note that the BaseScope probes were designed to hybridize to mRNA, thus yielding stronger signals for highly expressed (*TRB*) than lowly expressed (*TET2*) genes. (B) Double BaseScope ISH and immunohistochemistry demonstrate that both clonal T‐cell populations are CD4 positive, but CD8 negative.

To investigate the above speculation, we performed BaseScope ISH using specific probes targeting the unique TRB VDJ junctional sequence in two cases (one AITL and one PTCL‐TFH). As shown in case AITL030 (Figure 3), the V7‐J1 probe identified a diffuse cell population, while the V27‐J2 probe revealed only scattered cells, confirming that they represented two independent clonal T‐cell populations. To examine whether both clonal T‐cell populations carry the same *TET2* mutations, we further designed the BaseScope probes for the two *TET2* mutations in AITL030 and performed double BaseScope ISH by combining the TRB VDJ probe with each of the *TET2* mutation probes. The results showed co‐localization of the TRB‐VDJ and each of the two *TET2* mutation probe signal in both T‐cell populations (Figure 4A), indicating that both clonal T‐cell populations harboured the same *TET2* mutations.

To further investigate the immunophenotype, particularly that of the minor V27‐J2 clone, we combined BaseScope ISH with immunohistochemistry, and demonstrated that both of the clonal T‐cell populations are CD4 positive, but CD8 negative (Figure 4B).

### Biased *TRB* gene usage in AITL and PTCL‐TFH

**Figure 5:**
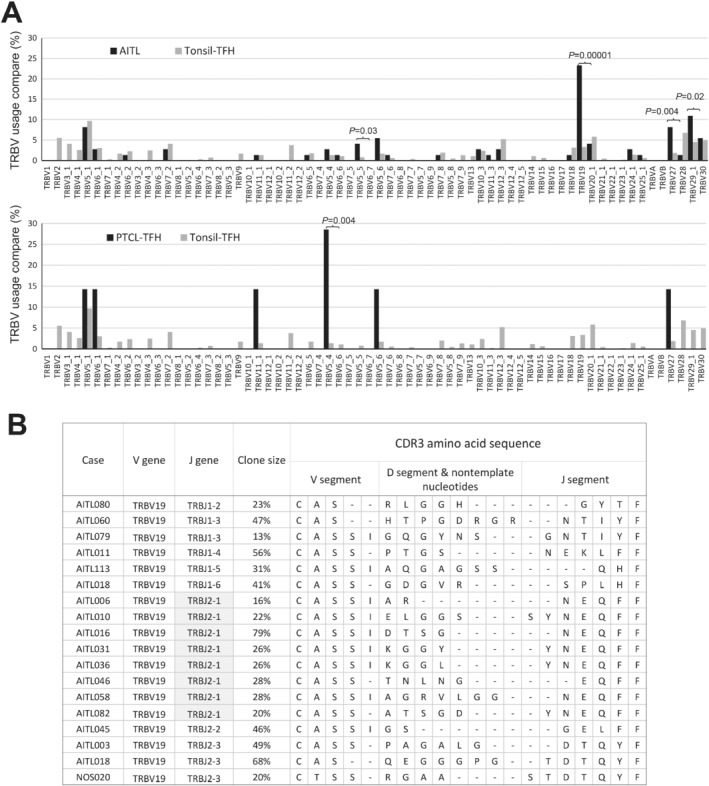
Biased usage of TRB variable genes in AITL and PTCL‐TFH. (A) Comparison of TRB variable gene usage between AITL/PTCL‐TFH and tonsillar TFH. (B) Comparison of CDR3 sequences among cases harbouring TRBV19 functional rearrangements. AITL031 and AITL036 have a nearly identical CDR3 sequence, suggesting possible recognition of a common antigen.

In comparison with human tonsillar TFH cells (1987 TRB rearrangements from three tonsils) 38, both AITL and PTCL‐TFH showed an over‐representation of several TRB variable family members, for example, TRBV19*01 in AITL, and TRBV27*01 in PTCL‐TFH (Figure 5A). Despite there were three TRBV19 polymorphic alleles, only TRBV19*01 was used in AITL. Among the 18 TRBV19*01 functional rearrangements seen in AITL and PTCL‐TFH, 8 involved TCR βJ2‐1. In two cases (AITL031 and AITL036), the VDJ junctional sequence was nearly identical with difference only at a single amino acid, strongly arguing for their similarity in antigen recognition (Figure 5B).

## Discussion

This study reports several novel findings that expand our knowledge on the pathological features of AITL and PTCL‐TFH, and also offer fresh insights into their multistage development and pathogenesis. We confirmed a similar mutation profile between AITL and PTCL‐TFH as demonstrated by previous studies 11, 17. We showed evidence of bi‐ or oligoclonal functional TRB rearrangements in 26% of these T‐cell lymphomas and confirm two independent clonal T‐cell populations for selected cases. We demonstrated that the two independent T‐cell clones in the same case share the same *TET2* mutations, suggesting derivation from a common precursor cell population. Finally, we found evidence of significantly biased usage of TRB variable genes in both AITL and PTCL‐TFH, with high identity of VDJ junctional sequence in some cases.

In the 2016 WHO lymphoma classification, a broad category of nodal lymphoma of follicular helper cell origin, including AITL, PTCL‐TFH, and follicular T‐cell lymphoma, was introduced due to their overlapping morphological, immunophenotypic, genetic, and clinical presentations, while also bearing certain distinct features. Several studies show that *RHOA* and *IDH2* mutations are strongly associated with the TFH immunophenotype and more extensive characteristics typical of AITL such as proliferation of follicular dendritic cells, high endothelial vessels, and presence of clear cells, with *IDH2* mutation defining a unique subgroup of AITL 12, 45, 46, 47. In support of these observations, we found *RHOA* mutation only in AITL and PTCL‐TFH but not in PTCL‐NOS, and *IDH2* mutation exclusively in AITL in the present study.

PCR analysis of the rearranged *TCR* genes is commonly used in aid of routine diagnosis of T‐cell lymphoma. However, the amplified PCR products are not routinely sequenced, thus not providing any definite data on the number of functional TCR rearrangements. By sequencing the PCR products, we showed evidence of bi‐ or oligo‐clonal functional TRB rearrangements in 26% of AITL and PTCL‐TFH. Although the clonal size could not be accurately measured by the adopted BIOMED‐2 assay as it was carried out in two different reaction tubes, there was a considerable difference in many cases between the clonal size of the different TRB rearrangements, with one being more predominant than the other, suggesting they may represent different T‐cell populations, rather than bi‐allelic rearrangements. This was confirmed by ISH using BaseScope probes to the unique TRB VDJ junctional sequence in selected cases.

**Figure 6:**
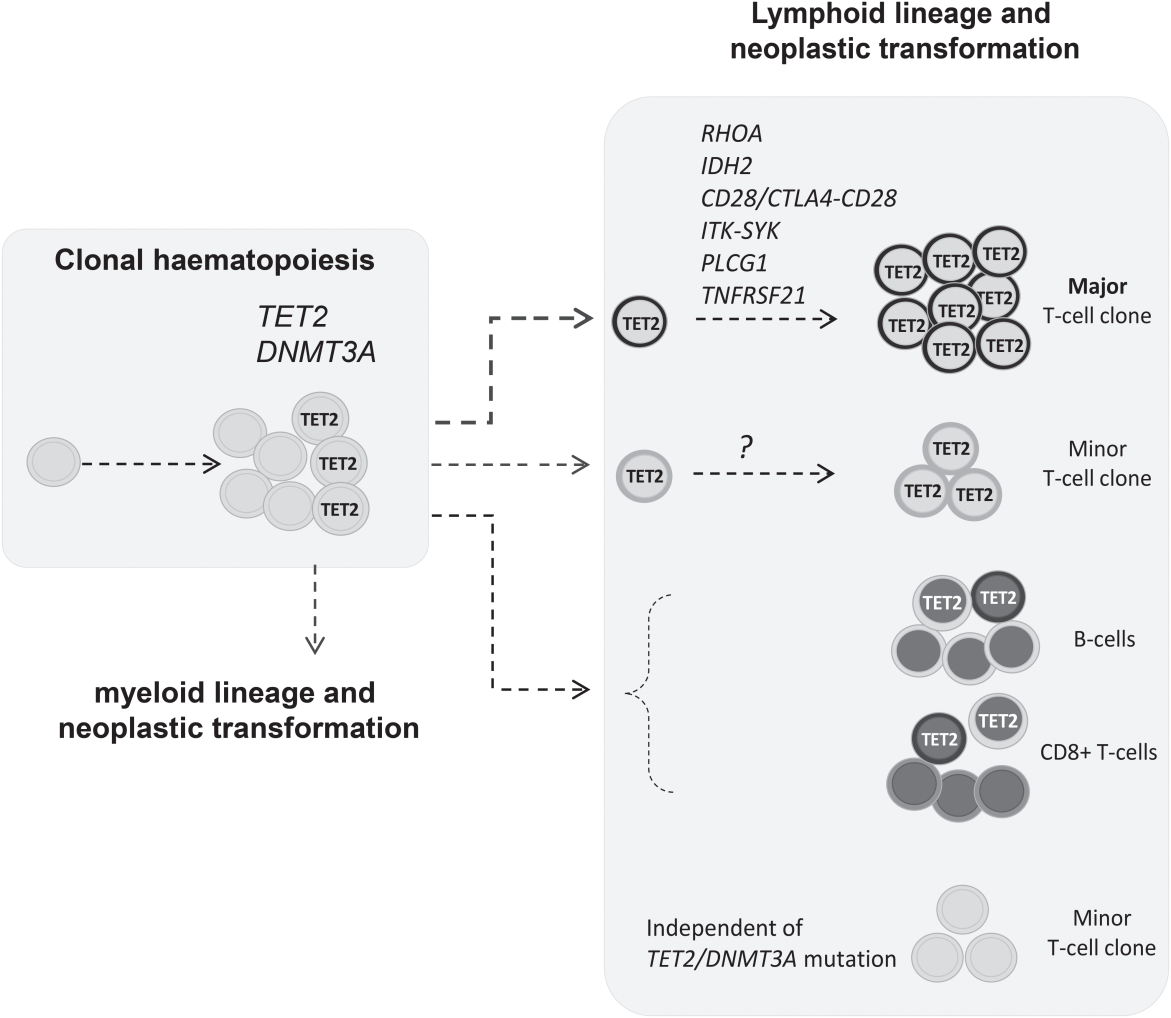
The proposed model of multistage development in AITL. Mutations in DNA methylation regulators—namely *TET2*, *DNMT3A*, and *IDH2*—are early events, with *TET2* and *DNMT3A* mutation occurring in hematopoietic stem cells. These mutations enhance the self‐renewal of haematopoietic stem cells, increase the risk of lymphomagenesis, and may frequently generate bi‐ or oligoclonal T‐cell populations in AITL or PTCL‐TFH following acquisition of additional genetic changes in genes important for T‐cell biology. The *TET2* mutant hematopoietic progenitor cells also give rise to non‐neoplastic polyclonal B and CD8 positive T‐cells as reported in several previous studies 7, 13, 14, 16, 21. Finally, there is also a possibility that minor T‐cell clones are not related to *TET2* or *DNMT3A* mutation but are the consequence of immunodeficiency and EBV reactivation in patients with AITL.

By using double BaseScope ISH, we were able to confirm that both clonal T‐cell populations carried the same *TET2* mutations in a representative case. These findings imply that these different T‐cell clones originated from a common precursor cell population that harboured the *TET2* mutations, and this common precursor cell population is most likely at a differentiation stage before *TCR* gene rearrangement. In patients with AITL, the lymphoma‐associated *TET2* mutations are frequently seen in haematopoietic progenitor cells and several lineages of non‐neoplastic cells including B and CD8+ T‐cells, which are polyclonal as reported to date 7, 13, 14, 16, 21. In this context, our finding of the lymphoma‐associated *TET2* mutation in an independent minor T‐cell population is not totally a surprise, but this raises an interesting question about whether the same *TET2* mutation may initiate an independent lymphomagenic process (Figure 6). Nonetheless, it remains to be investigated whether the above finding represents a common feature of AITL, or some of the minor T‐cell clones might be the consequence of immune reaction to immunodeficiency and EBV reactivation in patients with AITL, rather than due to *TET2* or *DNMT3A* mutation (Figure 6).

To investigate the above speculation, we performed double BaseScope ISH and immunohistochemistry, and demonstrated that both T‐cell populations in the index case are positive for CD4, but not CD8. Based on BaseScope ISH, the minor V27‐J2 clone was sparsely distributed in all the three independent tissue biopsies available for study, without showing any clusters. The nature and malignant potential of this minor T‐cell clone is unclear. Nonetheless, parallel malignant processes, such as development of AITL and myeloid leukaemia from the same *TET2* mutant haematopoietic progenitor population have been elegantly illustrated by recent case studies 48, 49.

The clinical relevance of the finding of an additional minor clonal T‐cell population in AITL and PTCL‐TFH remains to be investigated. It is essential to accurately determine the clonal size of such minor clonal T‐cell population and their evolution during disease progression using more established next‐generation sequencing––based TRB rearrangement analysis. In particular, it is important to investigate both metachronous and synchronous lesions to assess whether such minor clonal T‐cell population may become a major pathogenic player. This will help to identify suitable samples containing more expanded cell population for further in‐depth phenotypic and genetic investigations. Specimens differentially involved by distinct T‐cell clones would be highly valuable for further characterization of potential genetic changes in minor T‐cell clones, particularly *RHOA* and *IDH2* mutations, as they are frequent and specifically seen in the neoplastic T‐cell clone. Discrete T‐cells such as CD4+CD10+ T‐cells and CD8+ T‐cells could be isolated using fluorescence‐activated cell sorting and subjected to single‐cell analysis for *TCR* gene rearrangement and somatic mutation analysis.

Unlike *TET2* and *DNMT3A* mutations that occur at the haematopoietic stem cell level, both *RHOA* and *IDH2* mutations are restricted to the neoplastic T‐cell clone in AITL and PTCL‐TFH 7, 16, 21, 22. In line with this, both the *RHOA* and *IDH2* mutation loads were similar to each other, but significantly lower than the *TET2* and *DNMT3A* mutation load (supplementary material, Figure S3). In mouse model, RhoA G17 V cooperates with TCR stimulation to promote TFH‐cell expansion, and lymphoma development in absence of Tet2 30, 31, 50. The IDH2 R172 mutation causes a high level of oncometabolite 2‐hydroxyglutarate in the neoplastic T cells, which inhibits multiple α‐KG–dependent dioxygenases involved in various cell functions, including histone methylation and the hypoxia response 21. The frequent concurrence of both *RHOA* and *IDH2* mutations in AITL argues for their oncogenic synergy, and together they most likely play an important role in malignant transformation and also underpin the morphological and immunophenotypic presentations as discussed earlier.

By sequencing the rearranged *TCR* genes, we also demonstrated a significantly biased usage of certain TRB variable genes, particularly TRBV19*01 and TRBV27, in AITL and PTCL‐TFH. The high identify in the VDJ junctional sequence of TRBV19*01 rearrangement in two unrelated cases strongly argues for their similarity in antigen recognition. Although the antigen recognized by these TCRs and its impact on TCR signaling are unknown, a previous study demonstrated that among CD4+ T‐cells, those expressing TRBV19 or TRBV27 TCR were highly responsive to stimulation by staphylococcus enterotoxin B superantigen *in vitro* 51. Thus, it is pertinent to speculate that the intrinsic properties of TCR may render T cells to perpetual antigenic stimulation, chronic TCR signaling, hence clonal selection and expansion.

In summary, the present study shows evidence of bi‐ or oligoclonal T‐cell populations in a high proportion of AITL and PTCL‐TFH, and their derivation from a common *TET2* mutant progenitor cell population in a representative case (Figure 6). We also demonstrate a significant biased TRB variable gene usage in these T‐cell lymphomas, suggesting an important role of TCR signaling in clonal selection and expansion for malignant transformation.

## Author contributions statement

WQY and FW designed experiments, and collected and analysed data. LC carried out immunohistochemistry. SWZ, AC and MW designed the Fluidigm gene panel and provided technical assistance. JST and ZC carried out Illumina sequencing analysis and variant calling. HL, HB, YH, JWG, PW, HED, LRB, LF, GAF, ZG, ADA, SSC, MAK and WL contributed and reviewed cases. MQD, WY and FW wrote and prepared the manuscript. MQD designed and coordinated the study. All authors commented on the manuscript and approved its submission for publication.

## Supporting information


**Figure S1.** Nature and distribution of mutations in *TET2, DNMT3A, IDH2, RHOA, PLCG1, CCND3*, and *TNFRSF21* in AITL and PTCL‐TFH

**Figure S2.** Examples of somatic mutations identified by Fluidigm multiplex PCR and Illumina MiSeq sequencing in AITL

**Figure S3.** Comparison of mutation load among *TET2, DNMT3A, RHOA*, and *IDH2* changes

**Figure S4.** Examples of TRB sequencing by BIOMED‐2 PCR and Illumina MiSeq sequencing

**Table S1.** PCR primers and conditions used in the study

**Table S2.** List of mutations identified in AITL, PTCL‐TFH, and PTCL‐NOS
