# Supplementary material for: Angioimmunoblastic T‐cell lymphoma contains multiple clonal T‐cell populations derived from a common TET2 mutant progenitor cell
Source: J Pathol. 2020 Jan 16;250(3):346–57. doi: 10.1002/path.5376 (PMC7064999; doi:10.1002/path.5376)
Supplement: Supplementary file 1 — Figure S1. Nature and distribution of mutations in TET2, DNMT3A, IDH2, RHOA, PLCG1, CCND3, and TNFRSF21 in AITL and PTCL‐TFH [file PATH-250-346-s001.pdf]

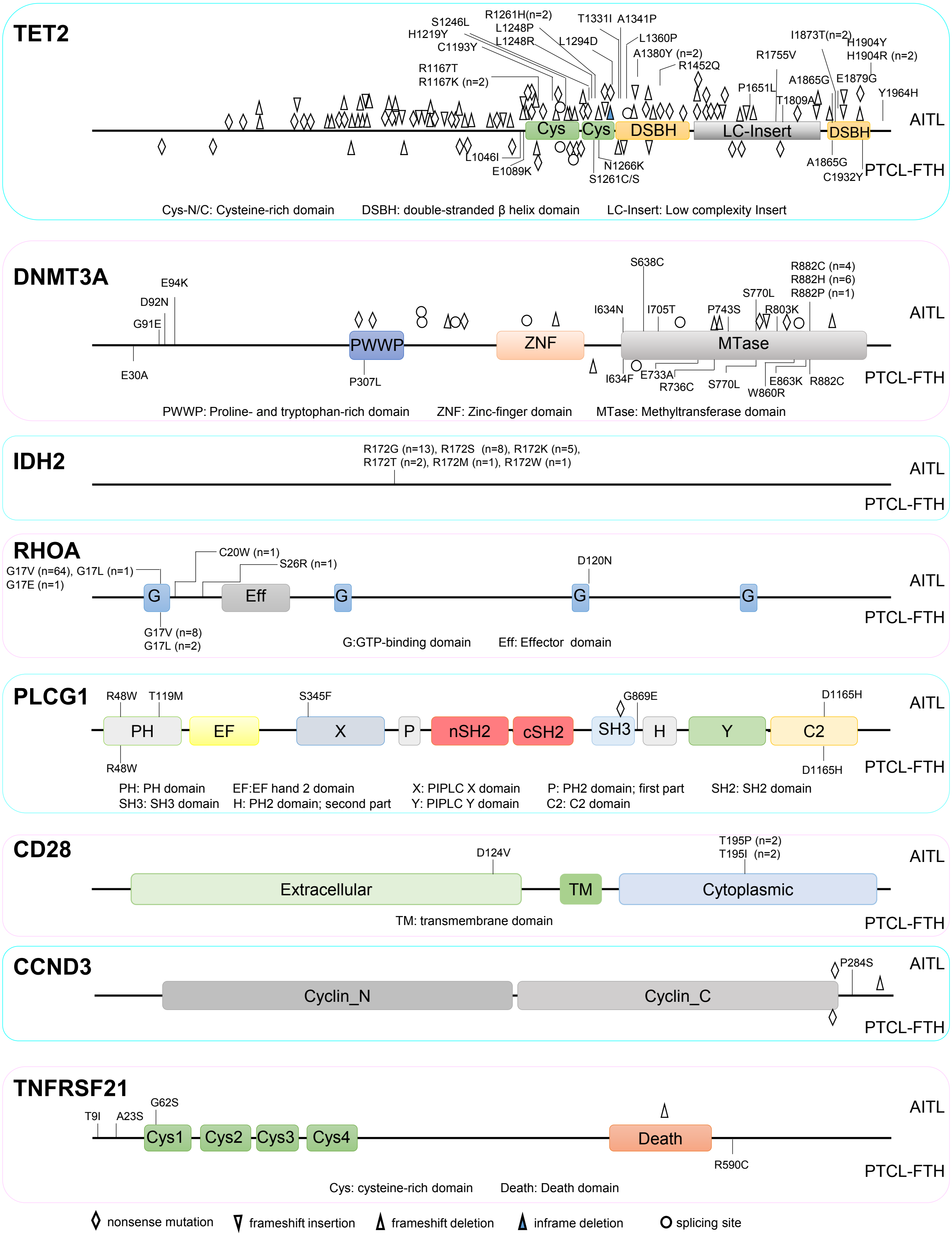

Figure S1. Nature and distribution of mutations in *TET2, DNMT3A, IDH2, RHOA, PLCG1, CCND3* and *TNFRSF21* in AITL and PTCL-TFH.
