# Supplementary material for: Angioimmunoblastic T‐cell lymphoma contains multiple clonal T‐cell populations derived from a common TET2 mutant progenitor cell
Source: J Pathol. 2020 Jan 16;250(3):346–57. doi: 10.1002/path.5376 (PMC7064999; doi:10.1002/path.5376)
Supplement: Supplementary file 2 — Figure S2. Examples of somatic mutations identified by Fluidigm multiplex PCR and Illumina MiSeq sequencing in AITL [file PATH-250-346-s002.pdf]

IDH2
AITL019
c.G516C
p.R172S

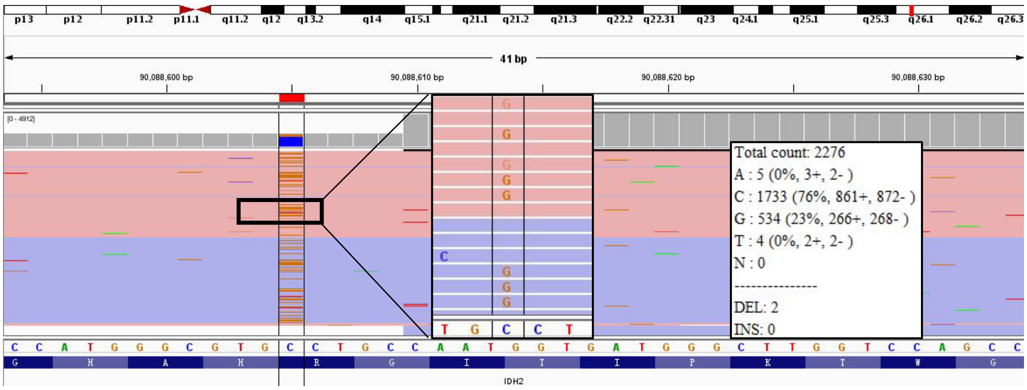

TET2
AITL065
c.A5636G
p.E1879G

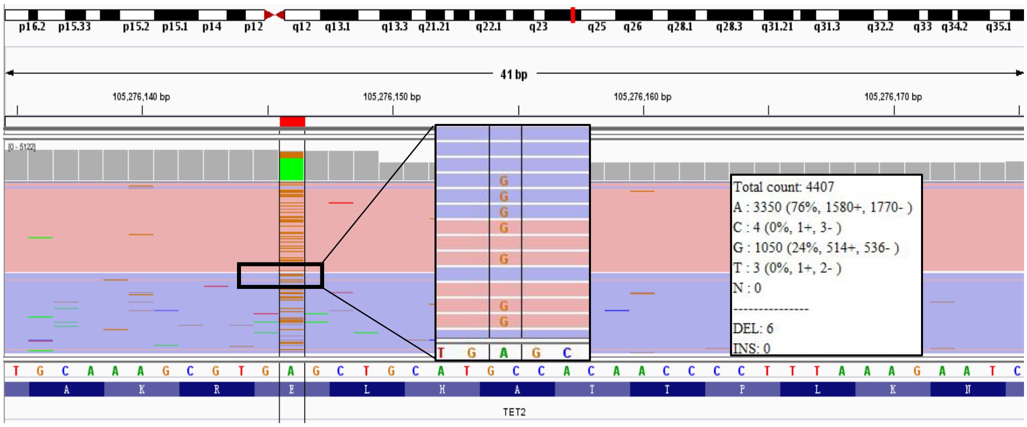

TET2
AITL063
c.2021_2028del
p.Q674fs

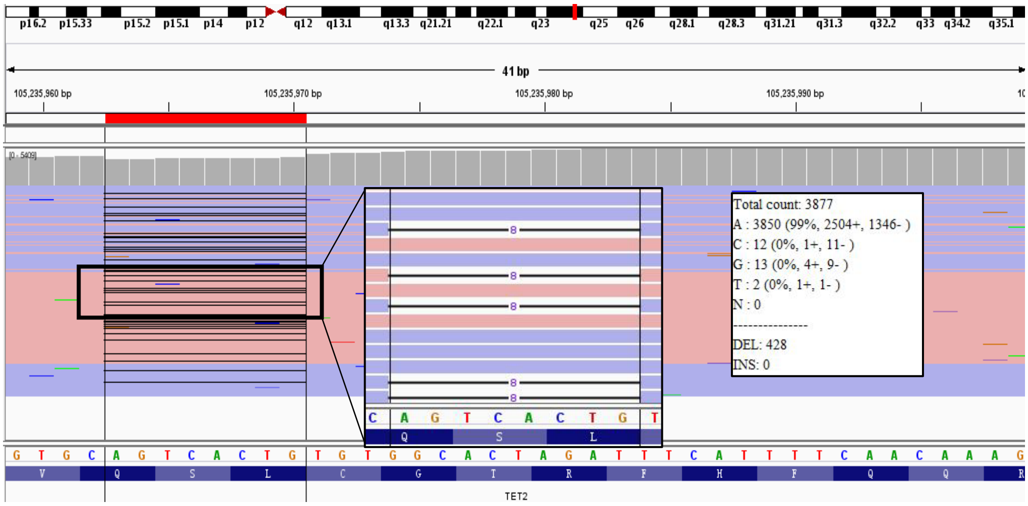

TET2
AITL043
c.1796delA
p.Q599fs

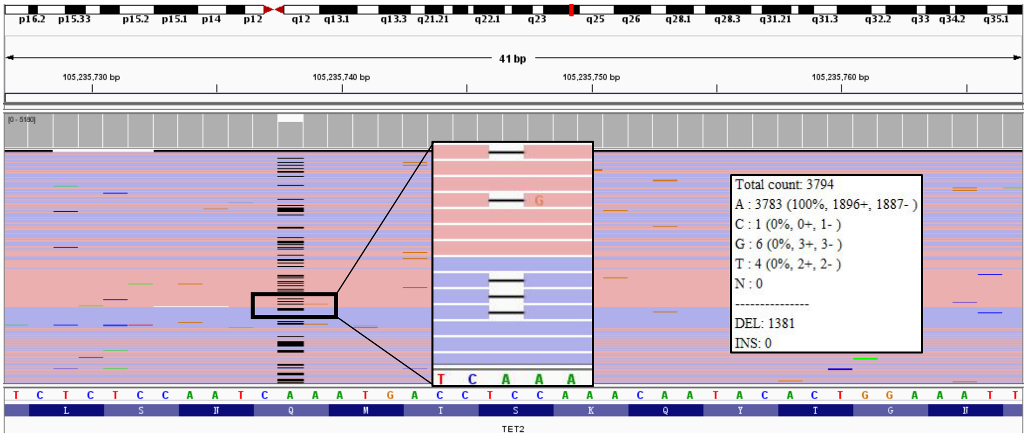

Figure S2: Examples of somatic mutations identified by Fluidigm multiplex PCR and Illumina MiSeq sequencing in AITL. . Aligned reads were transformed to a bam file and visualised using IGV software.
