# Supplementary figures and images for: Angioimmunoblastic T‐cell lymphoma contains multiple clonal T‐cell populations derived from a common TET2 mutant progenitor cell
Source: J Pathol. 2020 Jan 16;250(3):346–57. doi: 10.1002/path.5376 (PMC7064999; doi:10.1002/path.5376)

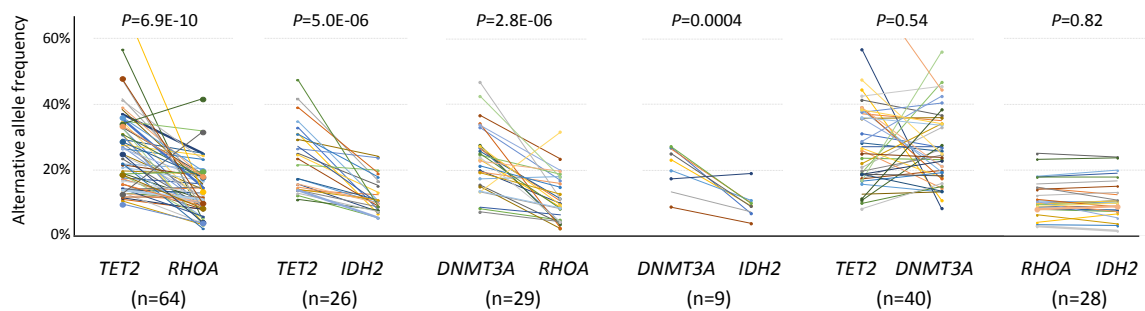

**Figure S3:** Comparison of mutation load among *TET2*, *DNMT3A*, *RHOA* and *IDH2* changes.

Supplement: Supplementary file 3 — Figure S3. Comparison of mutation load among TET2, DNMT3A, RHOA, and IDH2 changes [file PATH-250-346-s003.pdf]
