# Supplementary material for: Angioimmunoblastic T‐cell lymphoma contains multiple clonal T‐cell populations derived from a common TET2 mutant progenitor cell
Source: J Pathol. 2020 Jan 16;250(3):346–57. doi: 10.1002/path.5376 (PMC7064999; doi:10.1002/path.5376)
Supplement: Supplementary file 4 — Figure S4. Examples of TRB sequencing by BIOMED‐2 PCR and Illumina MiSeq sequencing [file PATH-250-346-s004.pdf]

AITL58
TRB-tube A

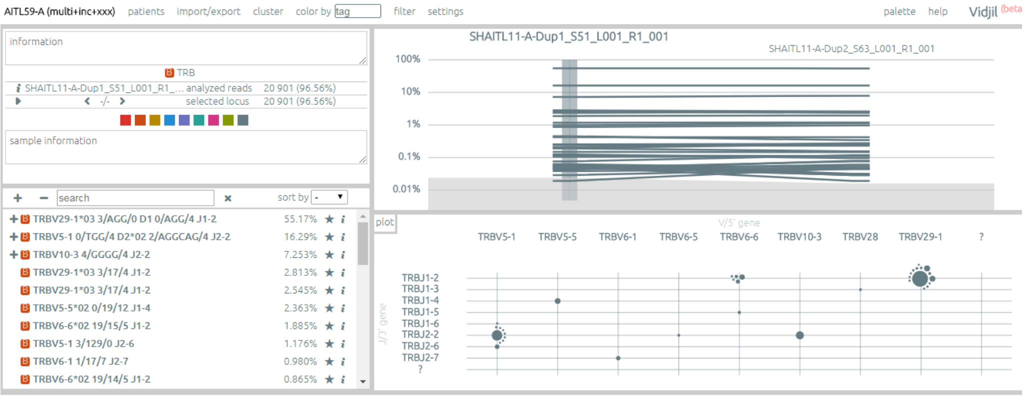

AITL58
TRB-tube B

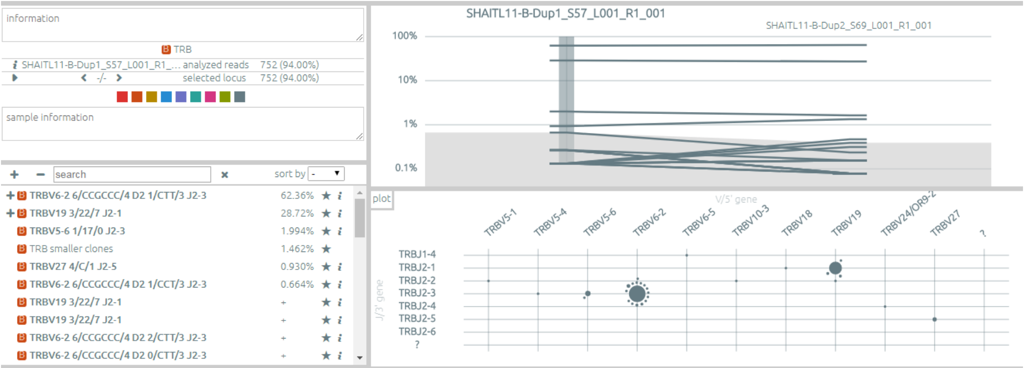

Figure S4: Examples of TRB sequencing by BIOMED-2 PCR and Illumina MiSeq sequencing. Sequence data were analysed using Vidjil software.
